# Supplementary material for: Design, Synthesis, and Anti-Melanogenic Activity of 2-Mercaptomethylbenzo[d]imidazole Derivatives Serving as Tyrosinase Inhibitors: An In Silico, In Vitro, and In Vivo Exploration
Source: Antioxidants (Basel). 2024 Oct 16;13(10):1248. doi: 10.3390/antiox13101248 (PMC11505594; doi:10.3390/antiox13101248)
Supplement: Supplementary file 1 [file antioxidants-13-01248-s001.zip › antioxidants-3254278-supplementary.pdf]

Supporting Information

For

Design, synthesis, and anti-melanogenic activity of 2-mercaptomethylbenzo[*d*]imidazole derivatives as tyrosinase inhibitors: In silico, in vitro and in vivo exploration

Hee Jin Jung [a,1], Hyeon Seo Park [a,1], Hye Jin Kim [a,1], Hye Soo Park [a,1], Yujin Park [b], Pusoon Chun [c], Hae Young Chung [d], Hyung Ryong Moon [a,*]

[a]*Department of Manufacturing Pharmacy, College of Pharmacy and Research Institute for Drug Development, Pusan National University, Busan 46241, Republic of Korea*

[b]*Department of Medicinal Chemistry, New Drug Development Center, Daegu-Gyeongbuk Medical Innovation Foundation, Daegu 41061, Republic of Korea*

[c]*College of Pharmacy and Inje Institute of Pharmaceutical Sciences and Research, Inje University, Gimhae 50834, Republic of Korea*

[d]*Department of Pharmacy, College of Pharmacy and Research Institute for Drug Development, Pusan National University, Busan 46241, Republic of Korea*

Contents

S1. ^{1}H NMR spectrum of analog **1** 4

S2. ^{13}C NMR spectrum of analog **1** 5

S3. ^{1}H NMR spectrum of analog **2** 6

S4. ^{13}C NMR spectrum of analog **2** 7

S5. ^{1}H NMR spectrum of analog **3** 8

S6. ^{13}C NMR spectrum of analog **3** 9

S7. ^{1}H NMR spectrum of analog **4** 10

S8. ^{13}C NMR spectrum of analog **4** 11

S9. ^{1}H NMR spectrum of analog **5** 12

S10. ^{13}C NMR spectrum of analog **5** 13

S11. ^{1}H NMR spectrum of analog **6**.......... 14

S12. ^{13}C NMR spectrum of analog **6** 15

S13. ^{1}H NMR spectrum of analog **7** 16

S14. ^{13}C NMR spectrum of analog **7** 17

S15. ^{1}H NMR spectrum of analog **8** 18

S16. ^{13}C NMR spectrum of analog **8** 19

S17. ^{1}H NMR spectrum of analog **9** 20

S18. ^{13}C NMR spectrum of analog **9** 21

S19. ^{1}H NMR spectrum of analog **10** 22

S20. ^{13}C NMR spectrum of analog **10** 23

S21. ^{1}H NMR spectrum of analog **11**.......... 24

S22. ^{13}C NMR spectrum of analog **11**.......... 25

S23. Alignment of the re-docked ligand (green) and co-crystallized ligand (red) with the 2Y9X protein..........26

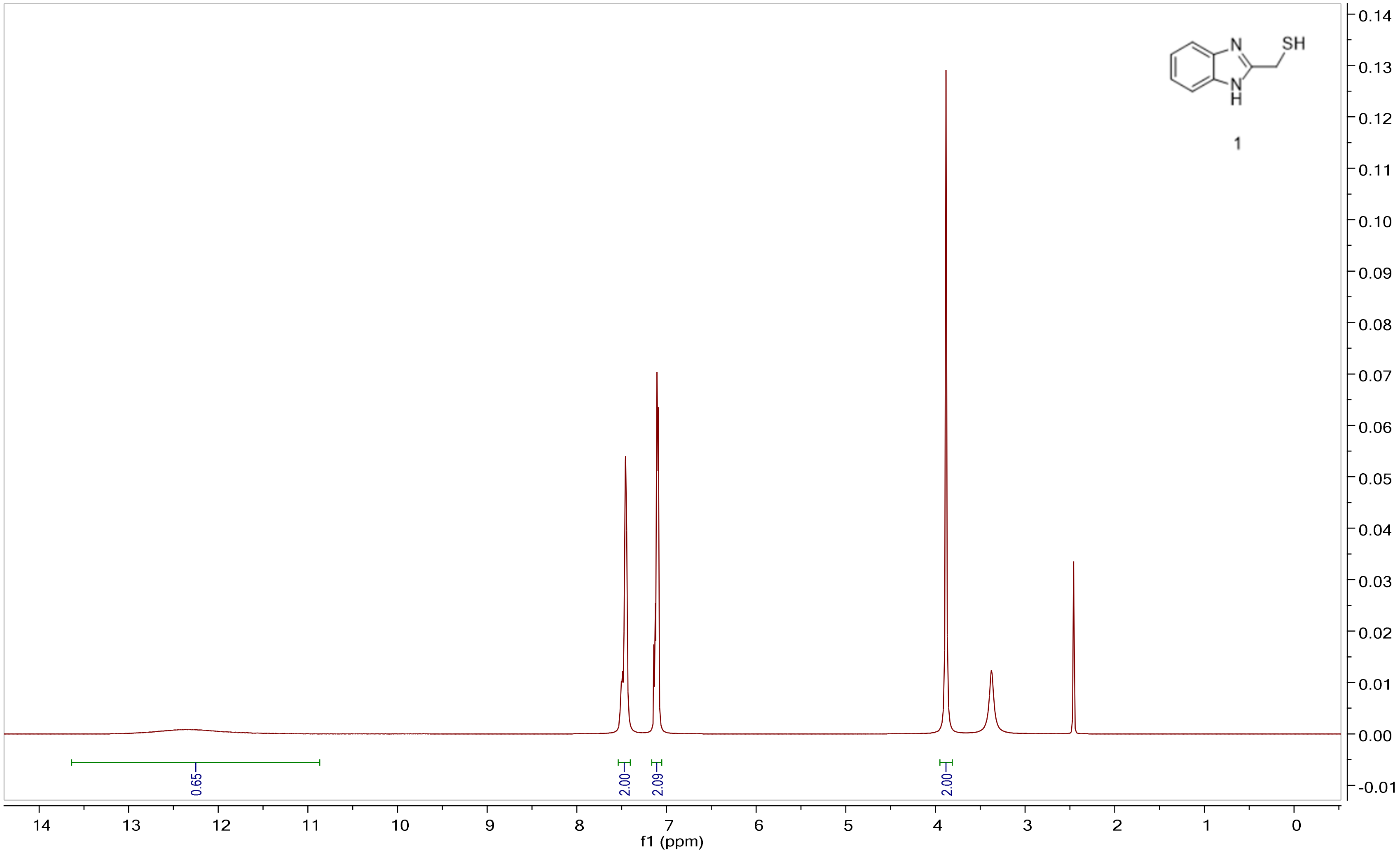

S1. ^{1}H NMR spectrum of analog **1**

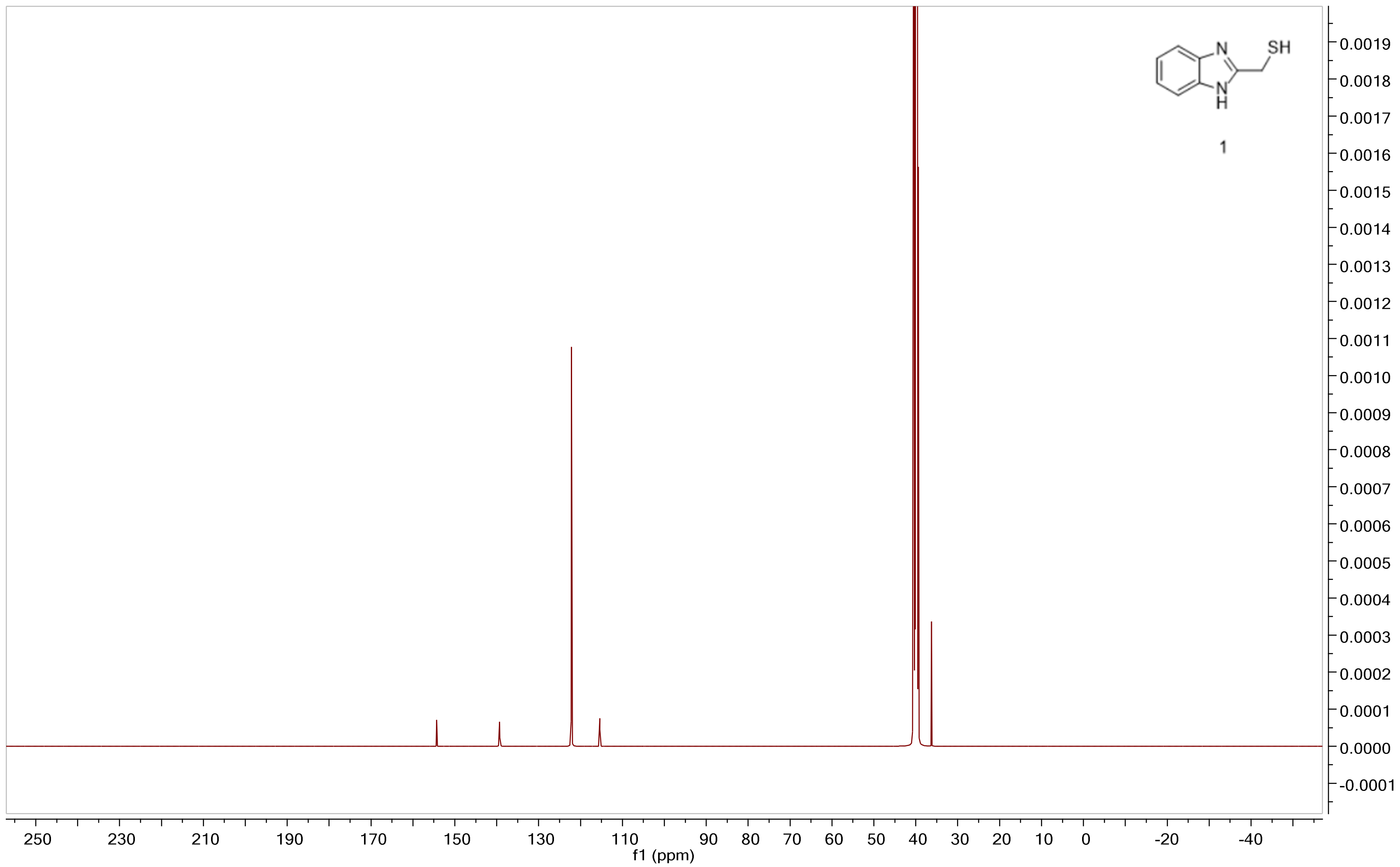

S2. ^{13}C NMR spectrum of analog **1**

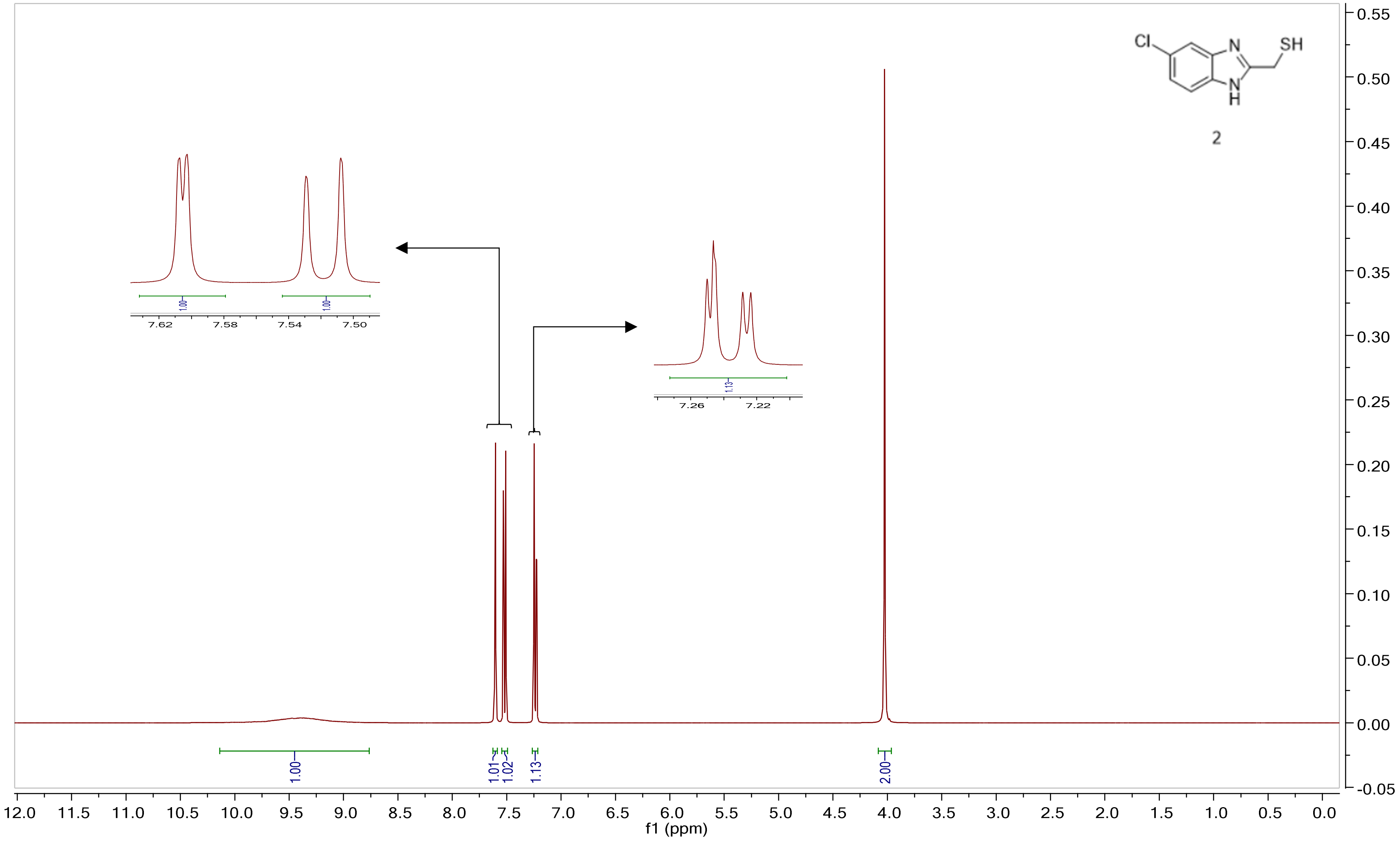

S3. ^{1}H NMR spectrum of analog **2**

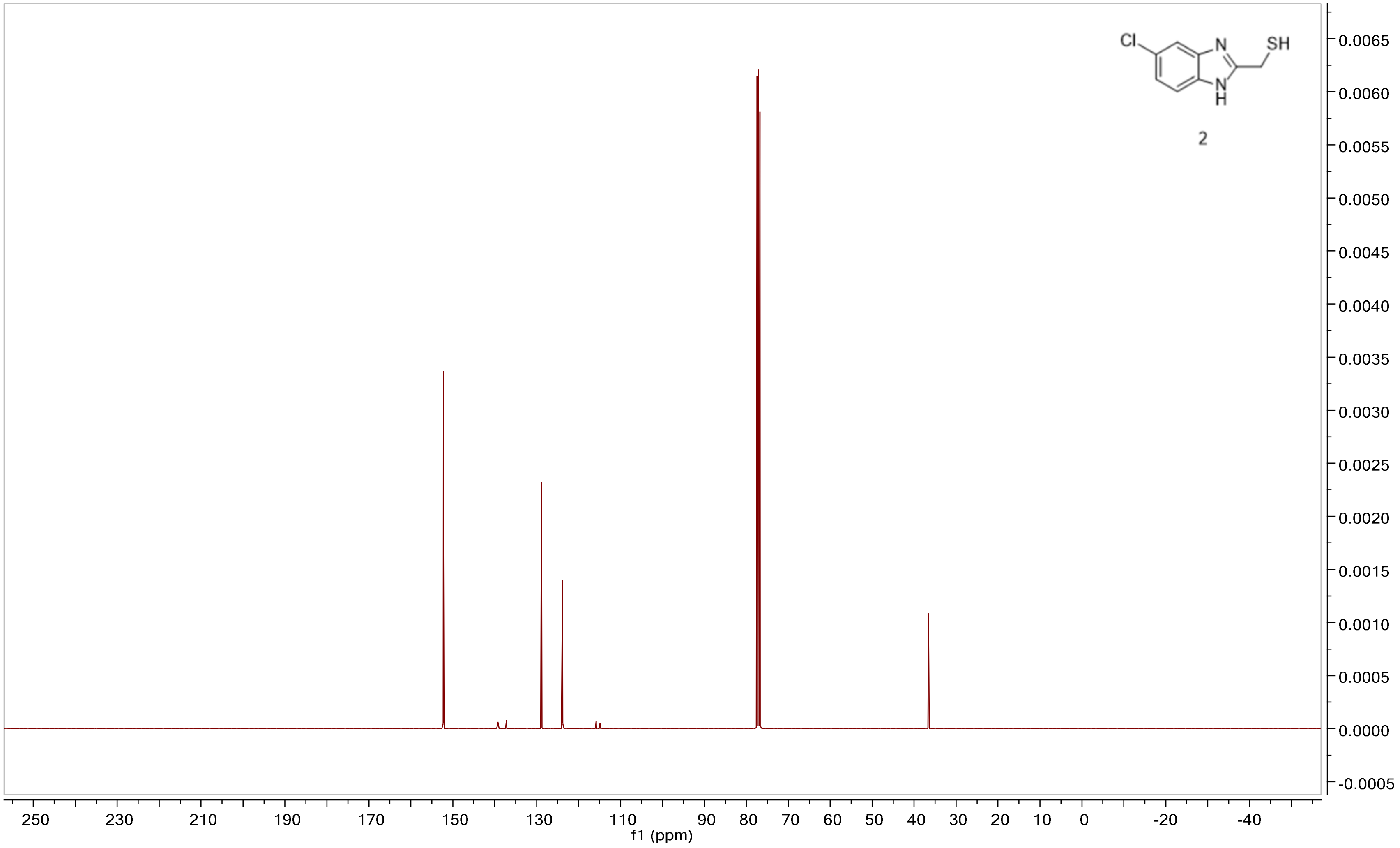

S4. ^{13}C NMR spectrum of analog **2**

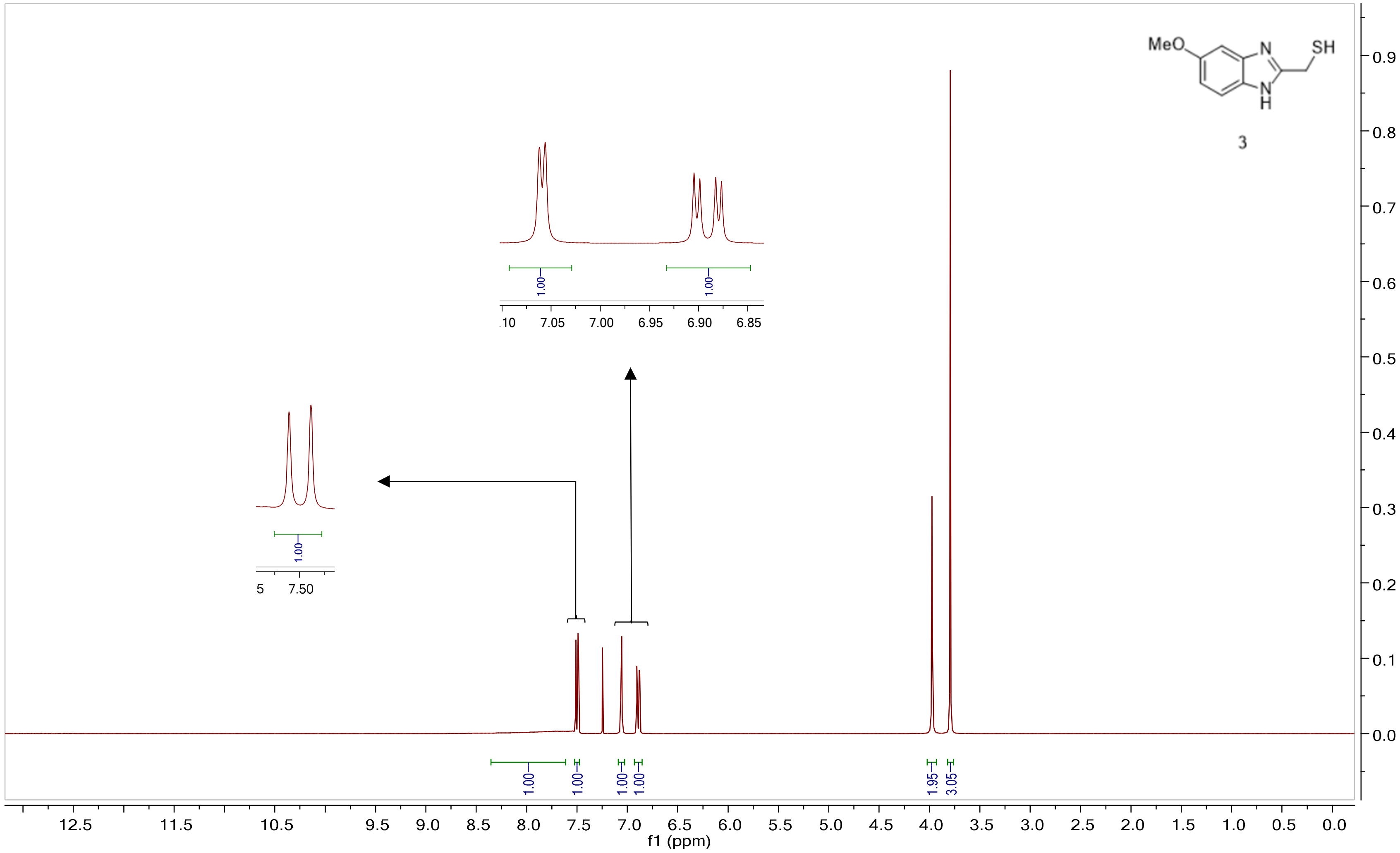

S5. ^{1}H NMR spectrum of analog **3**

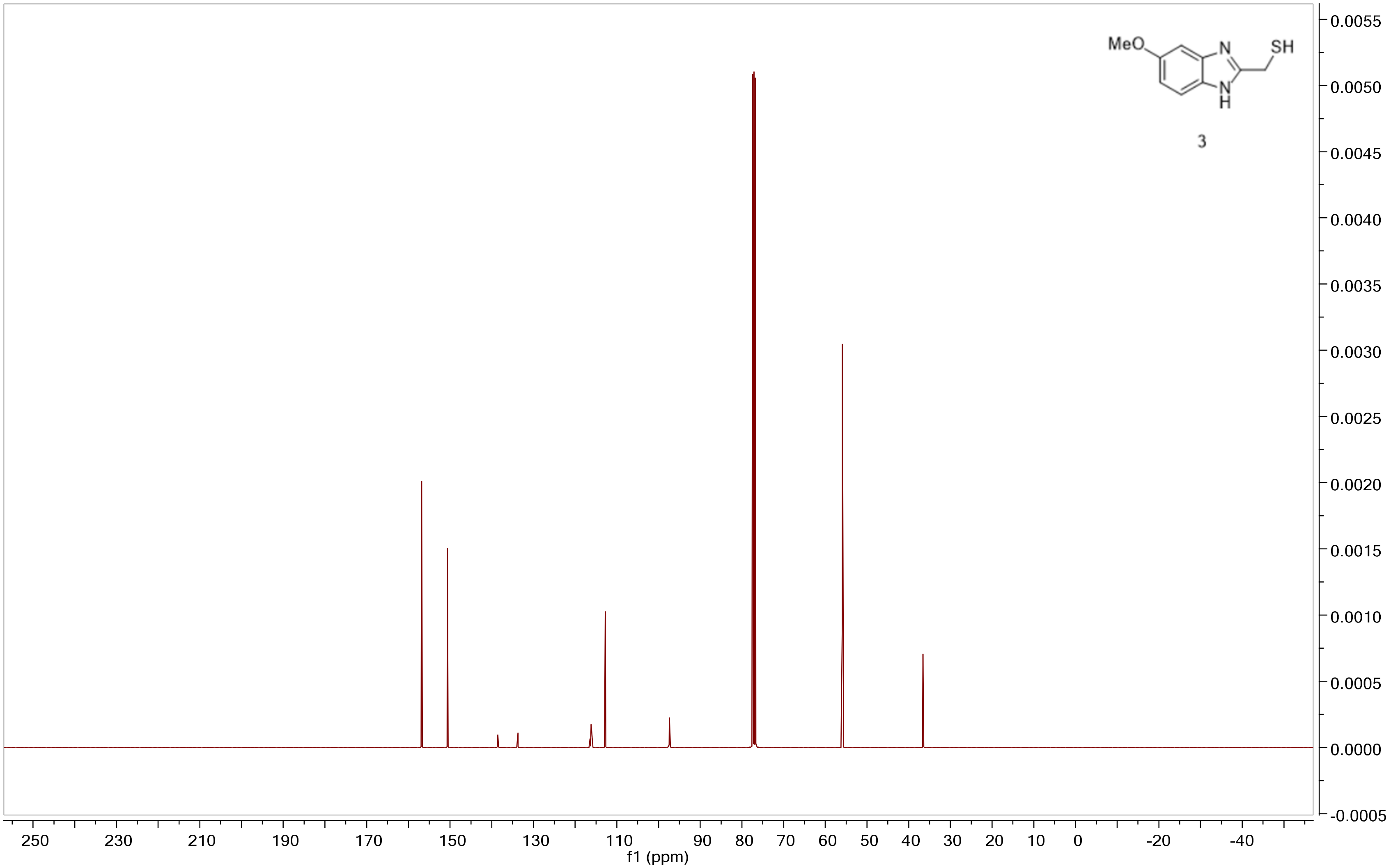

S6. ^{13}C NMR spectrum of analog **3**

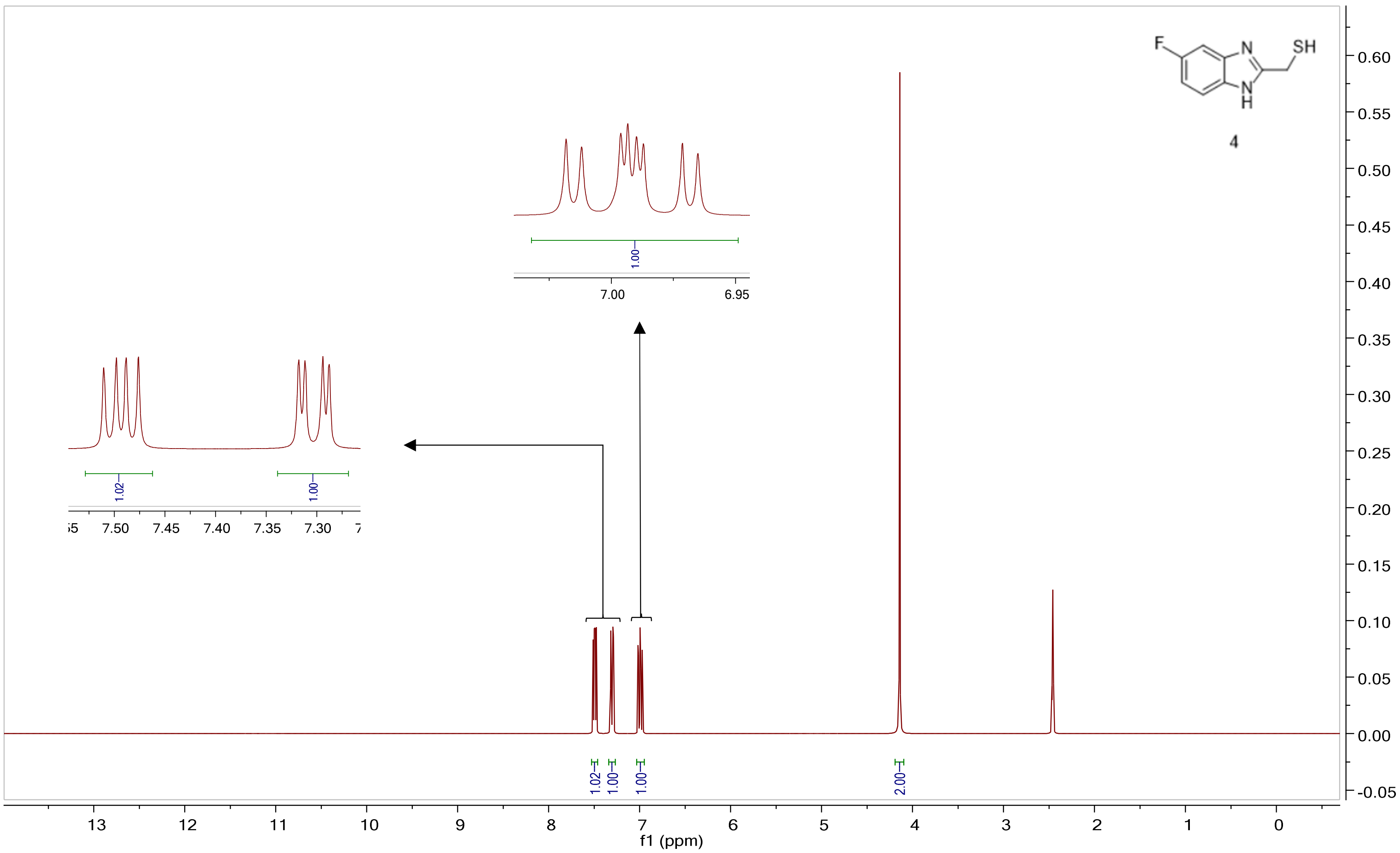

S7. ^{1}H NMR spectrum of analog **4**

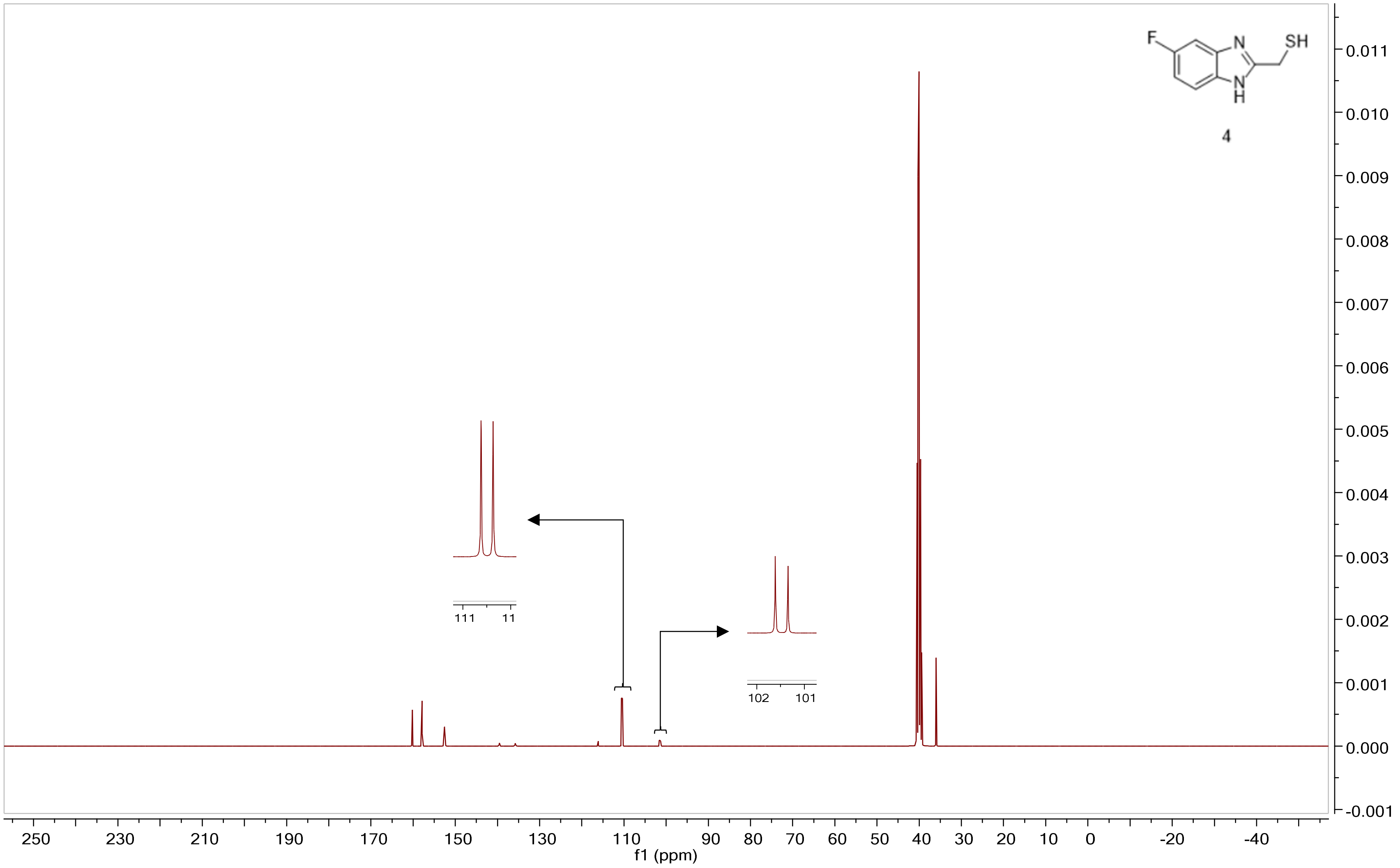

S8. ^{13}C NMR spectrum of analog **4**

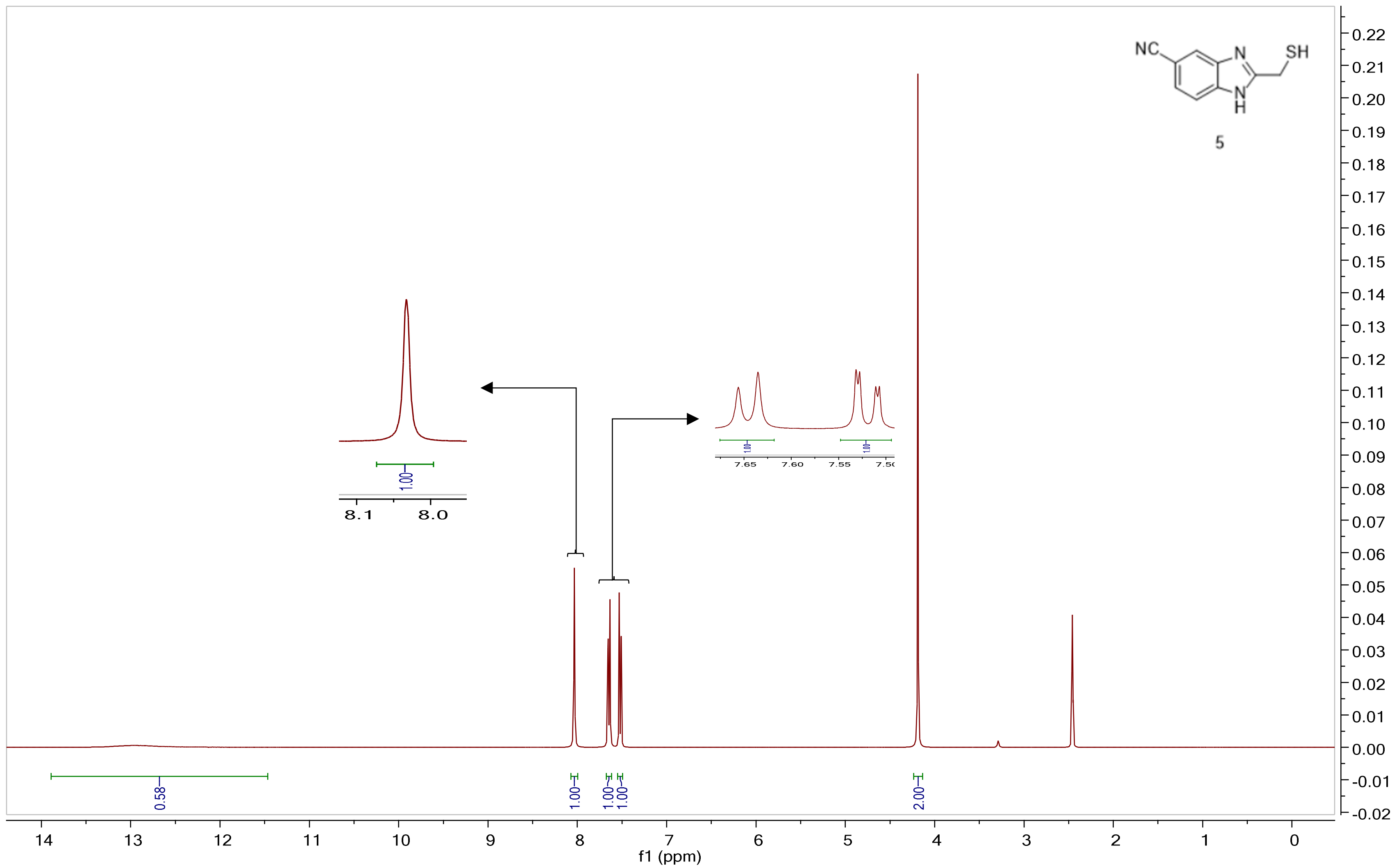

S9. ^{1}H NMR spectrum of analog **5**

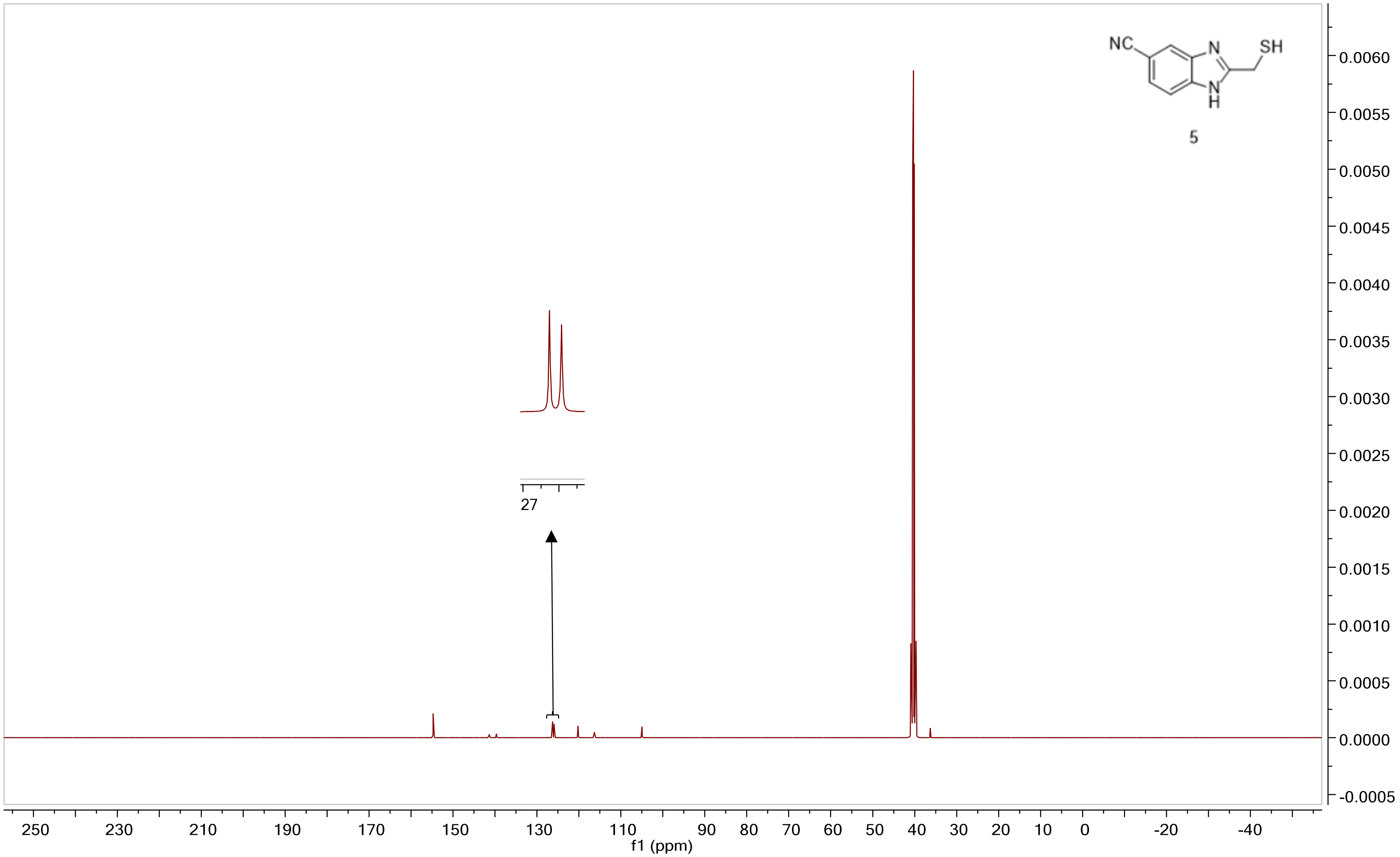

S10. ^{13}C NMR spectrum of analog **5**

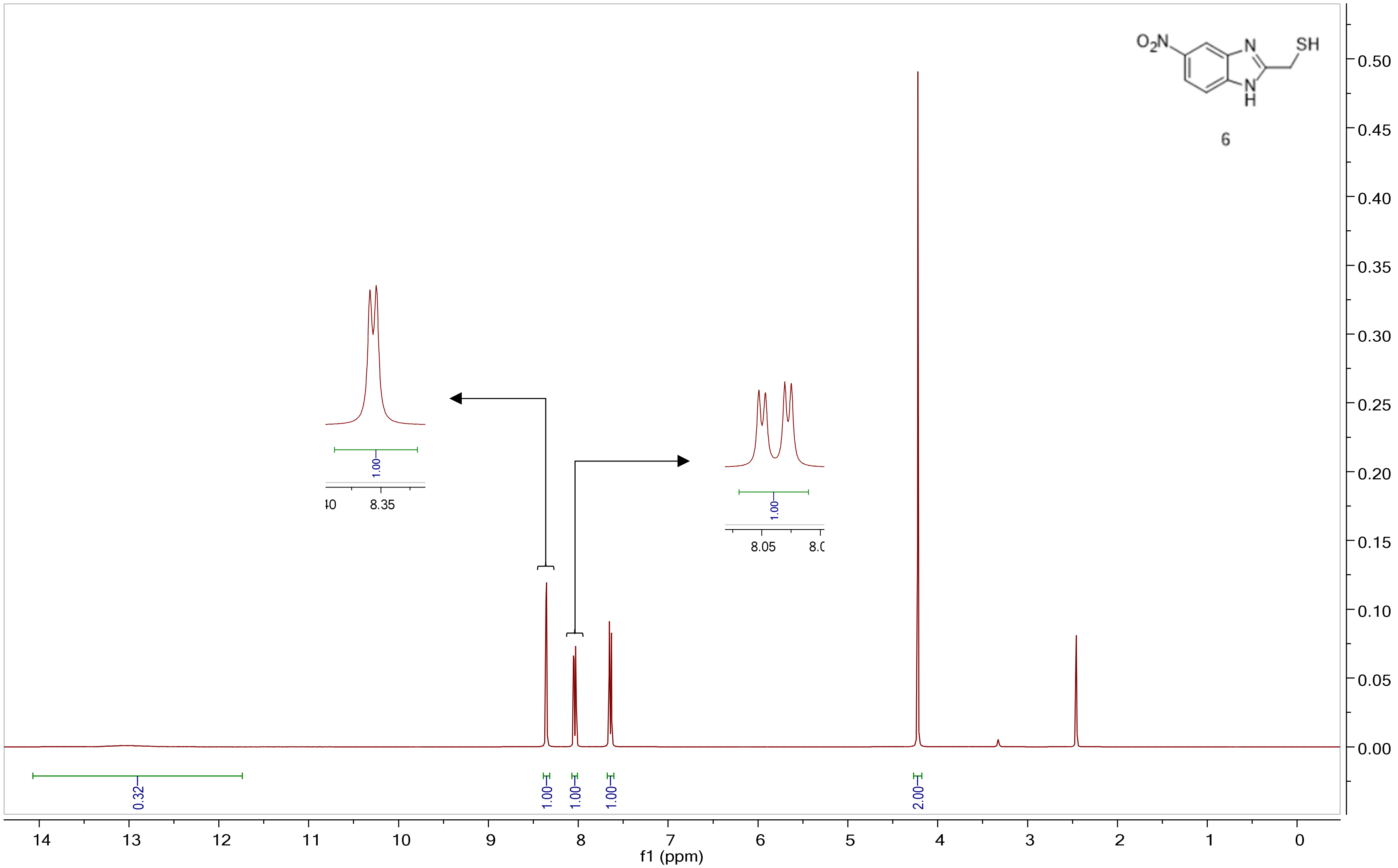

S11. ^{1}H NMR spectrum of analog **6**

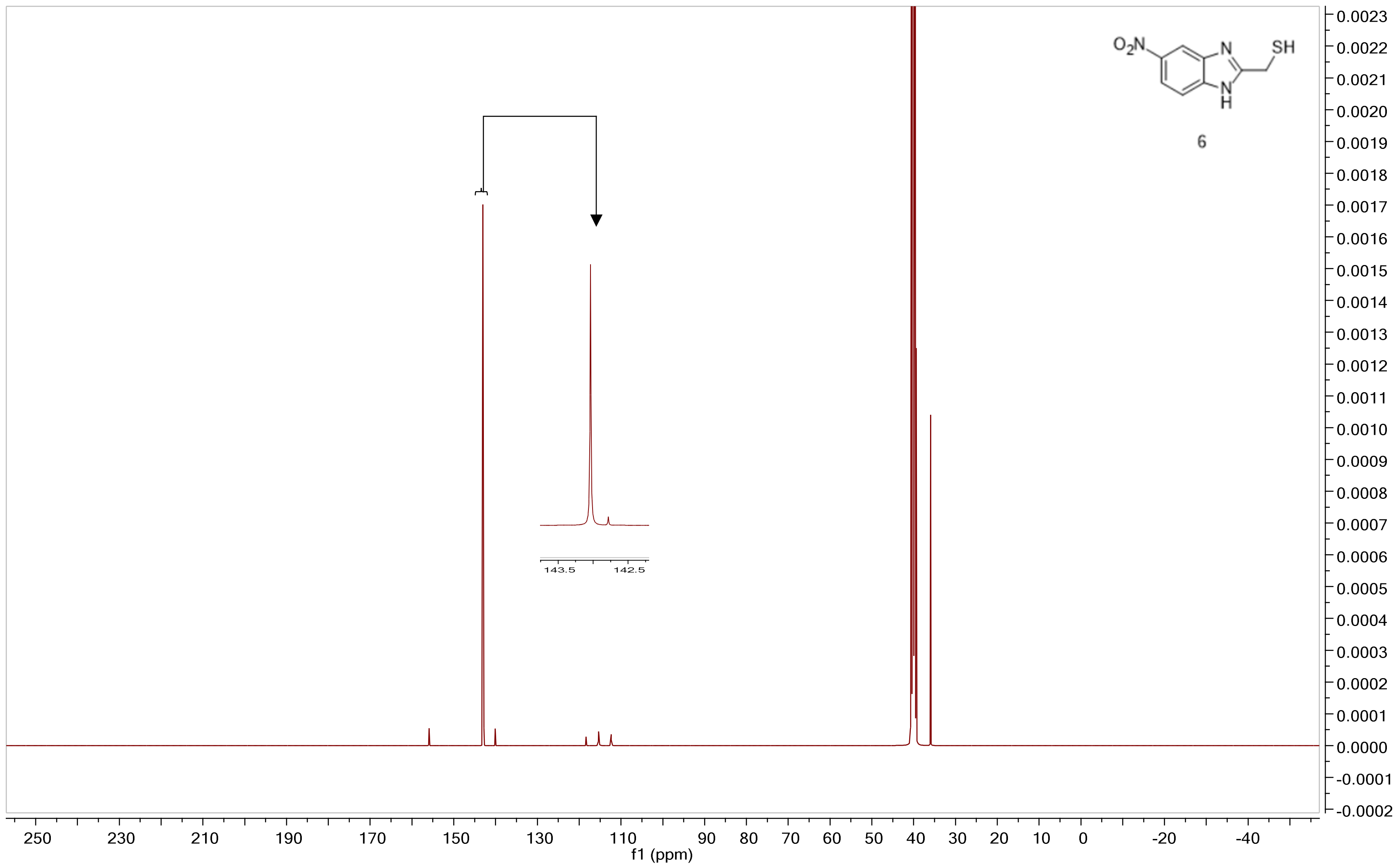

S12. ^{13}C NMR spectrum of analog **6**

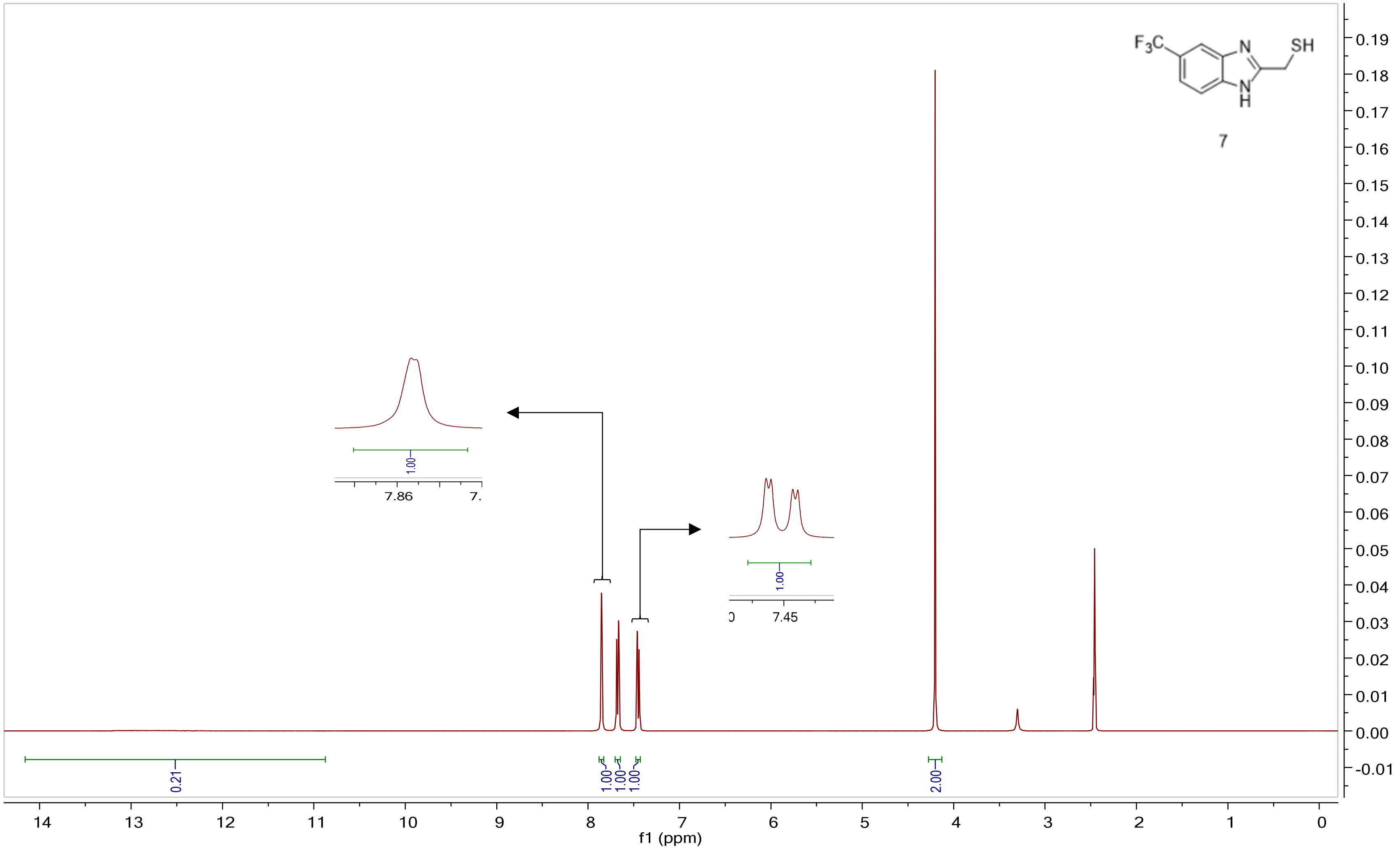

S13. ^{1}H NMR spectrum of analog **7**

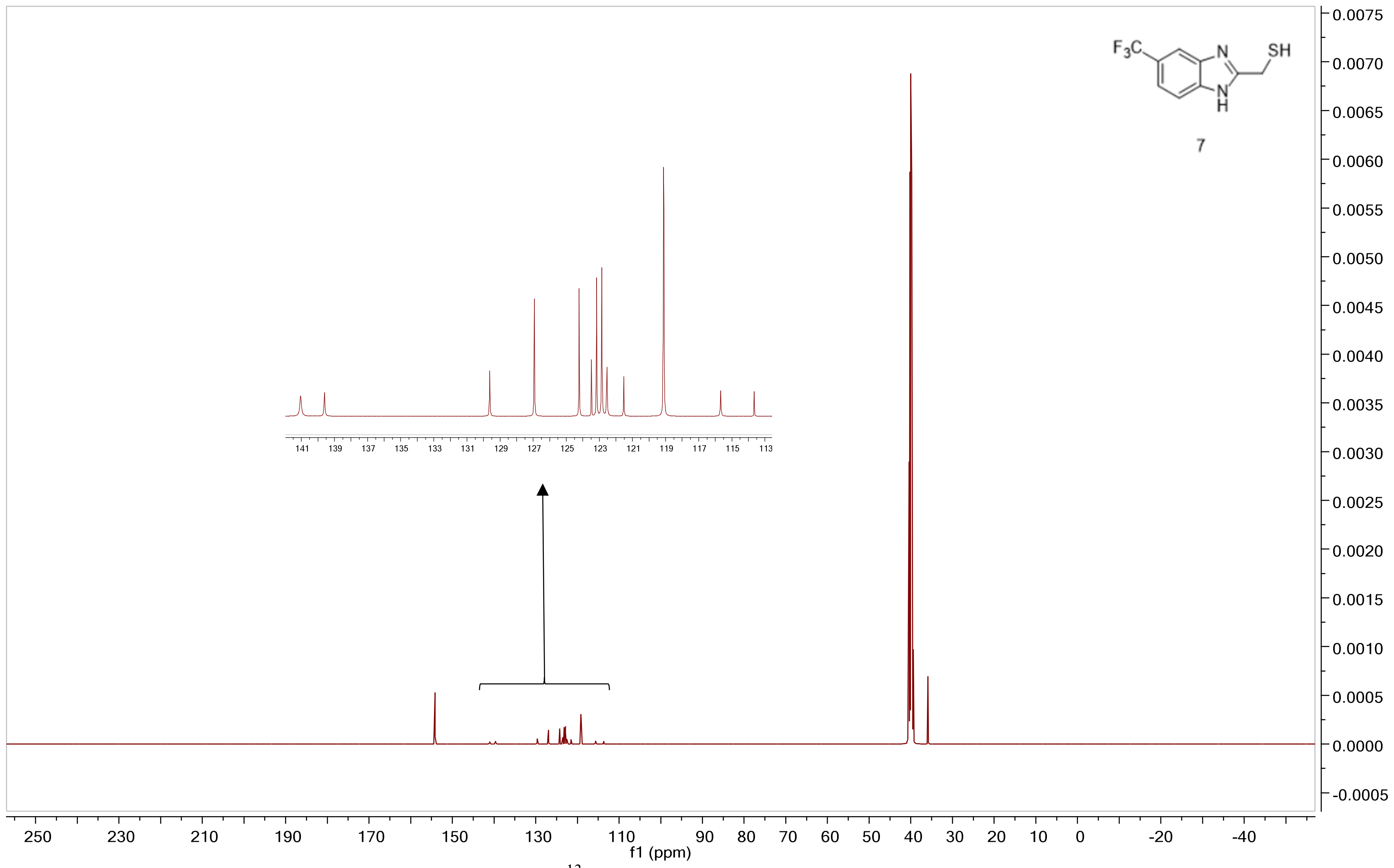

S14. ^{13}C NMR spectrum of analog **7**

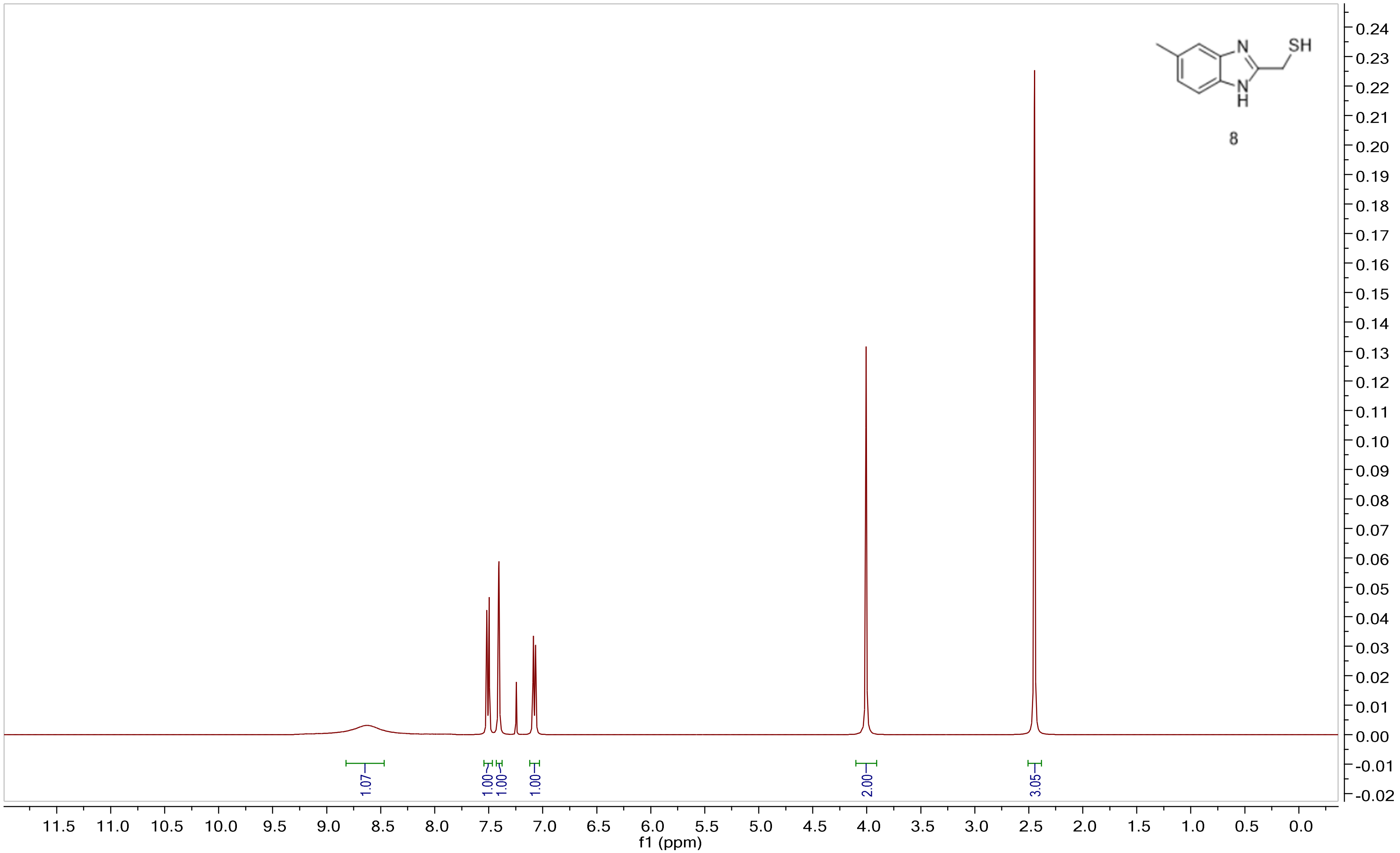

S15. ^{1}H NMR spectrum of analog **8**

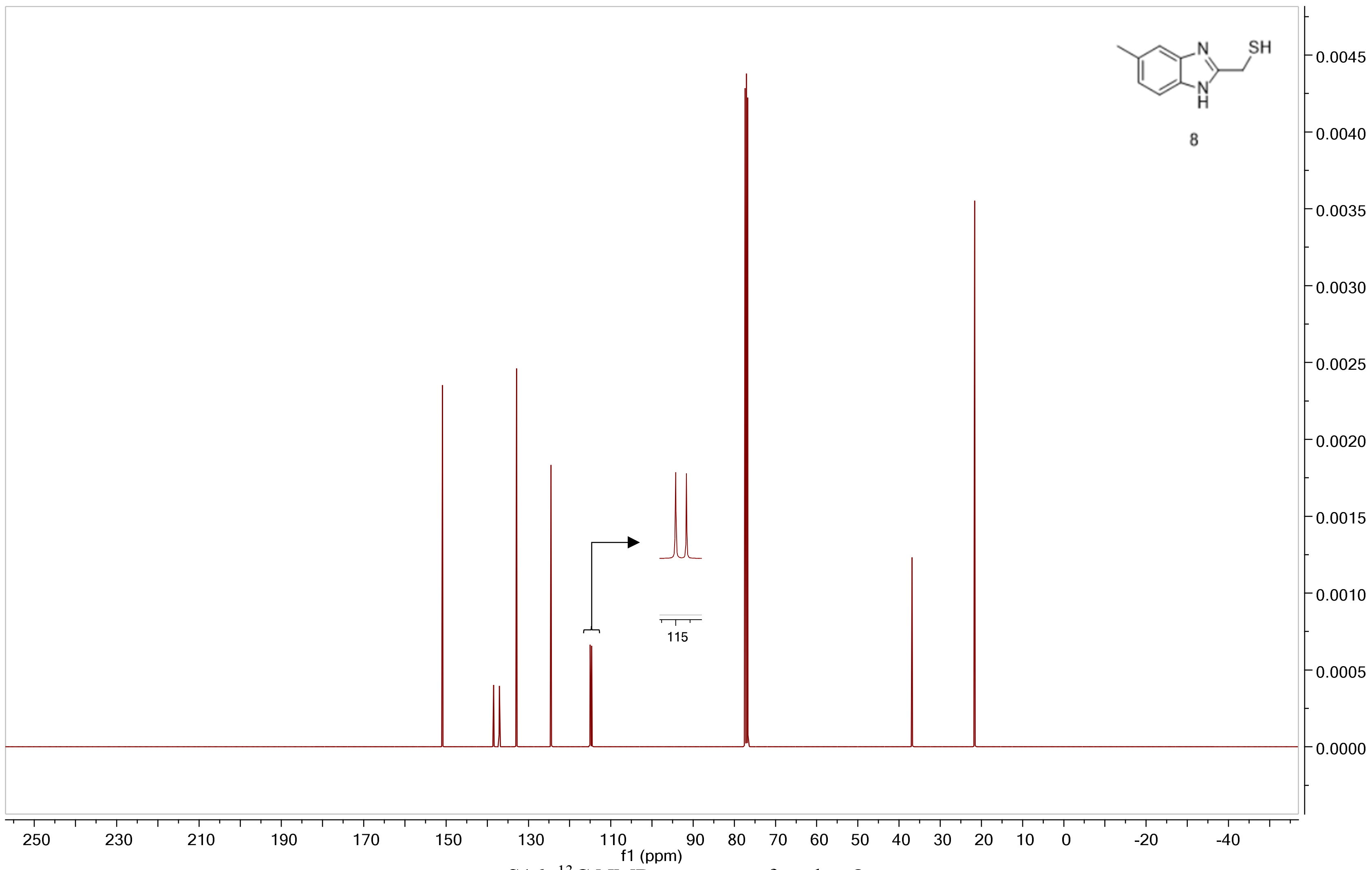

S16. ^{13}C NMR spectrum of analog **8**

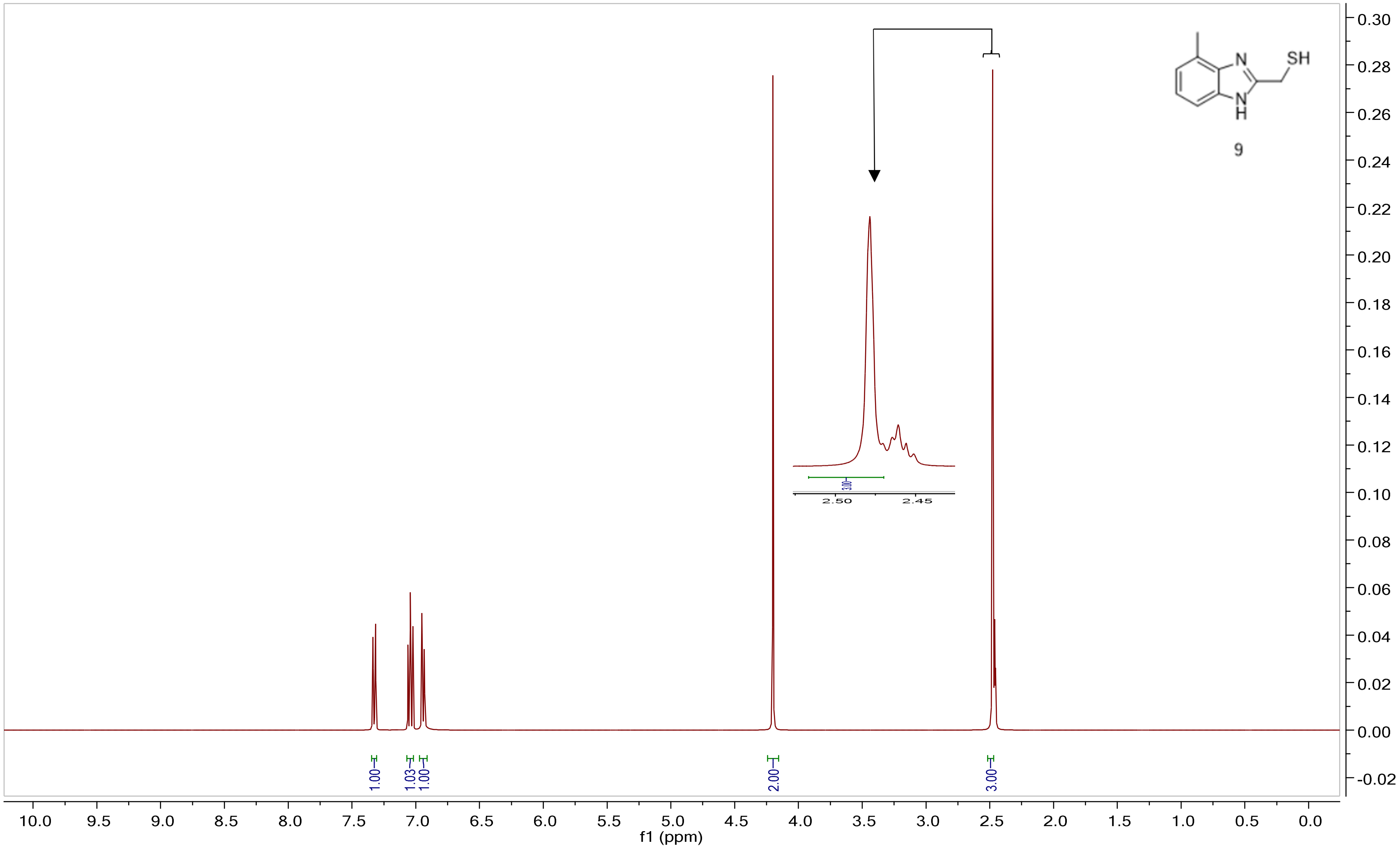

S17. ^{1}H NMR spectrum of analog **9**

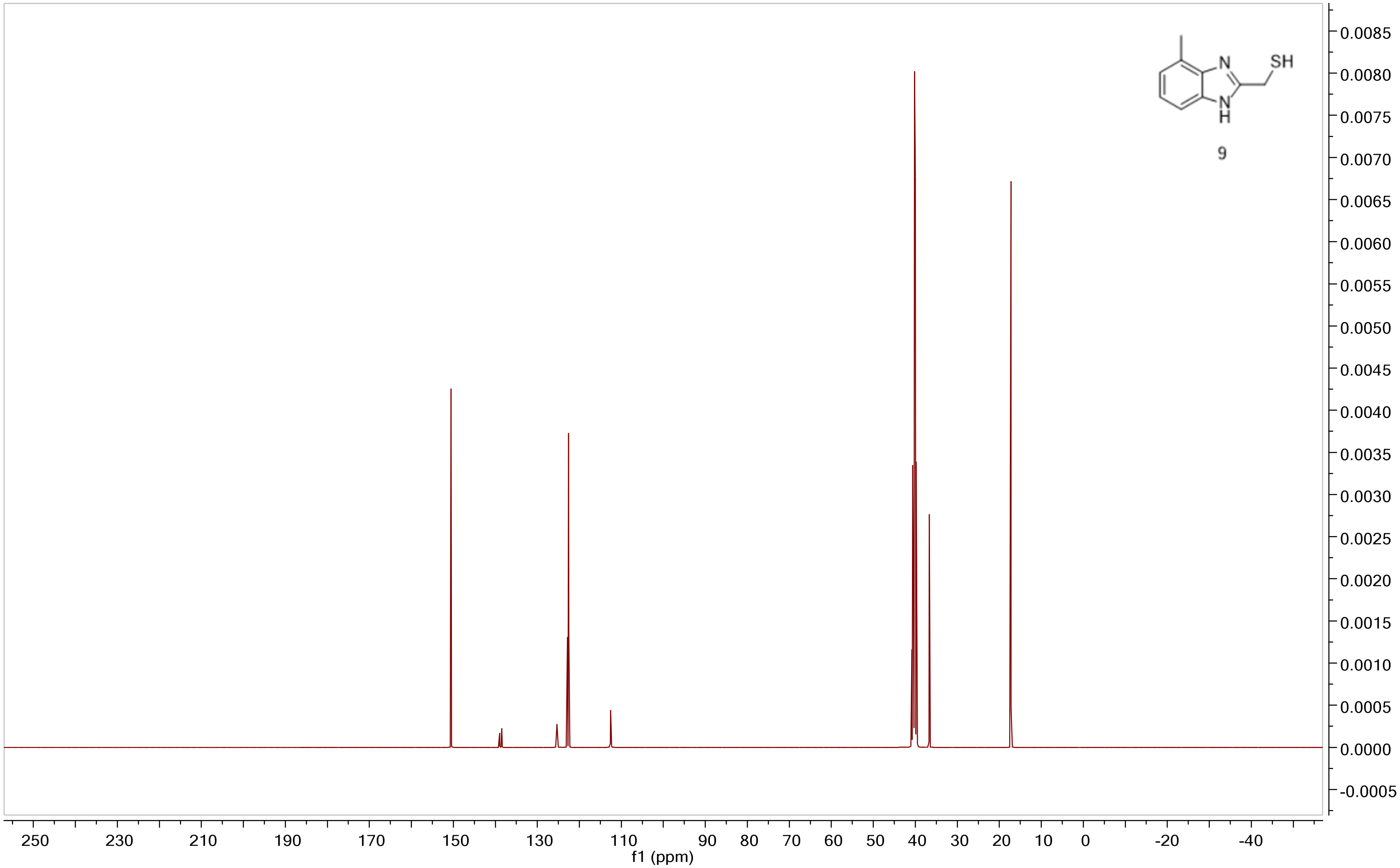

S18. ^{13}C NMR spectrum of analog **9**

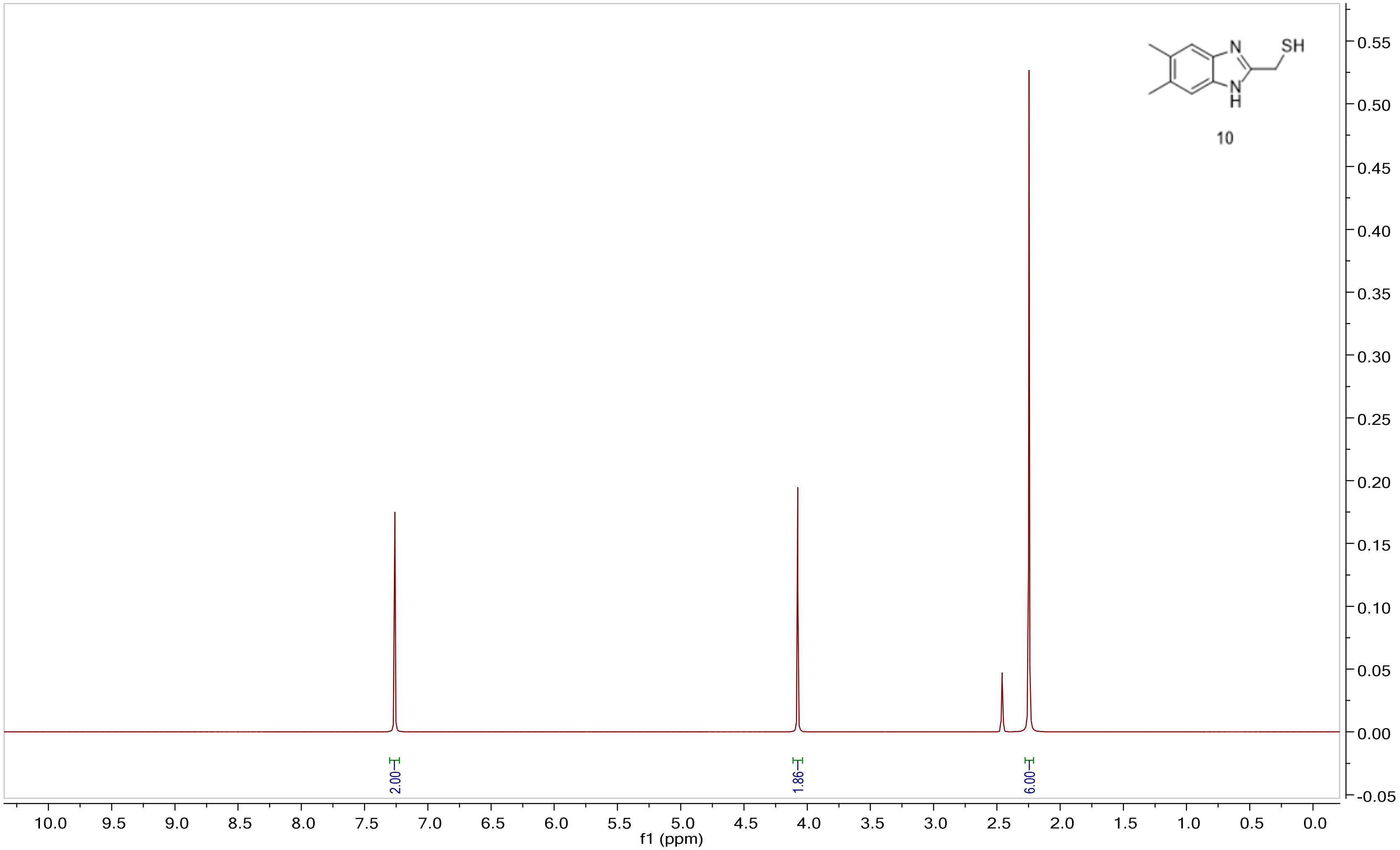

S19. ^{1}H NMR spectrum of analog **10**

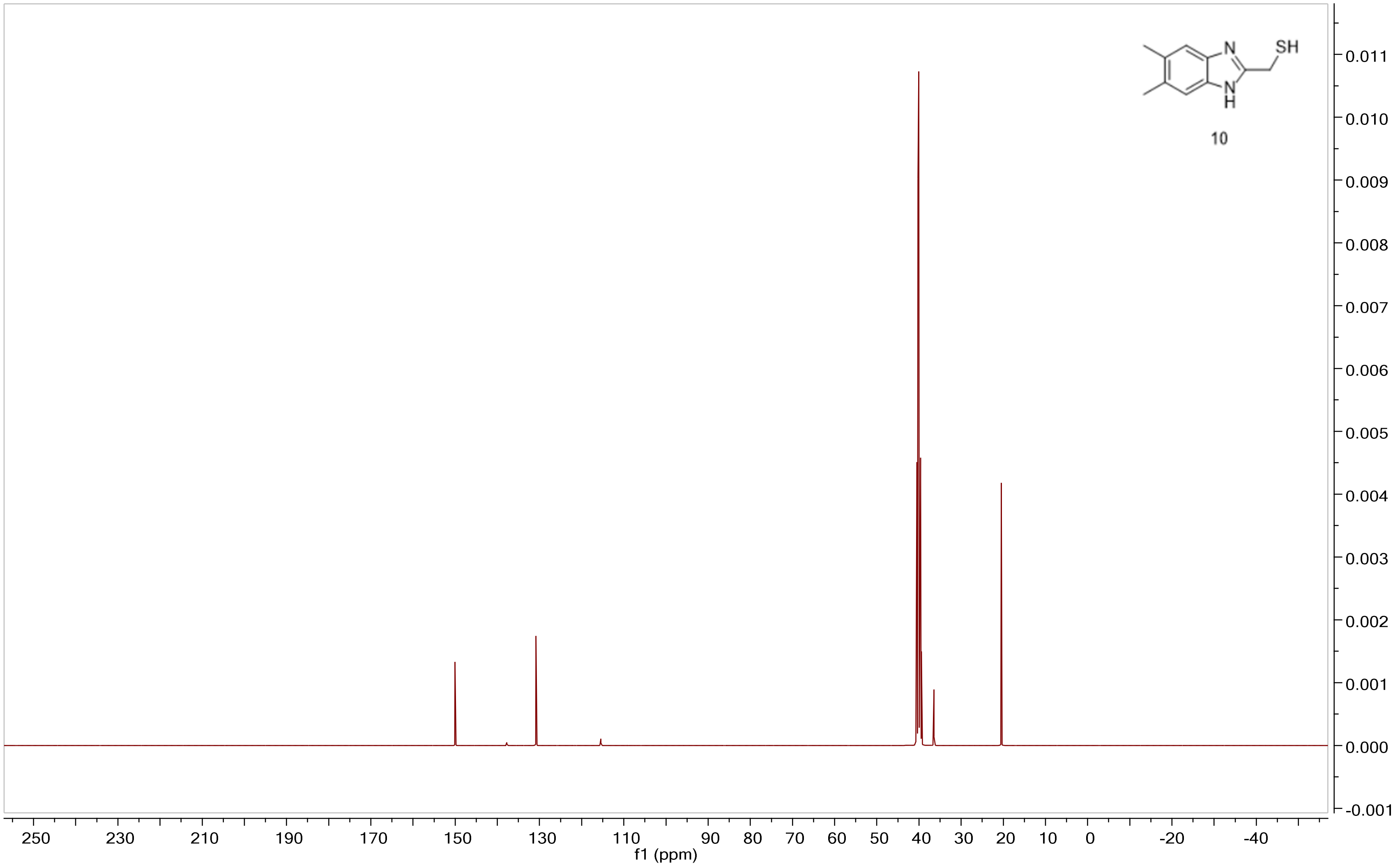

S20. ^{13}C NMR spectrum of analog **10**

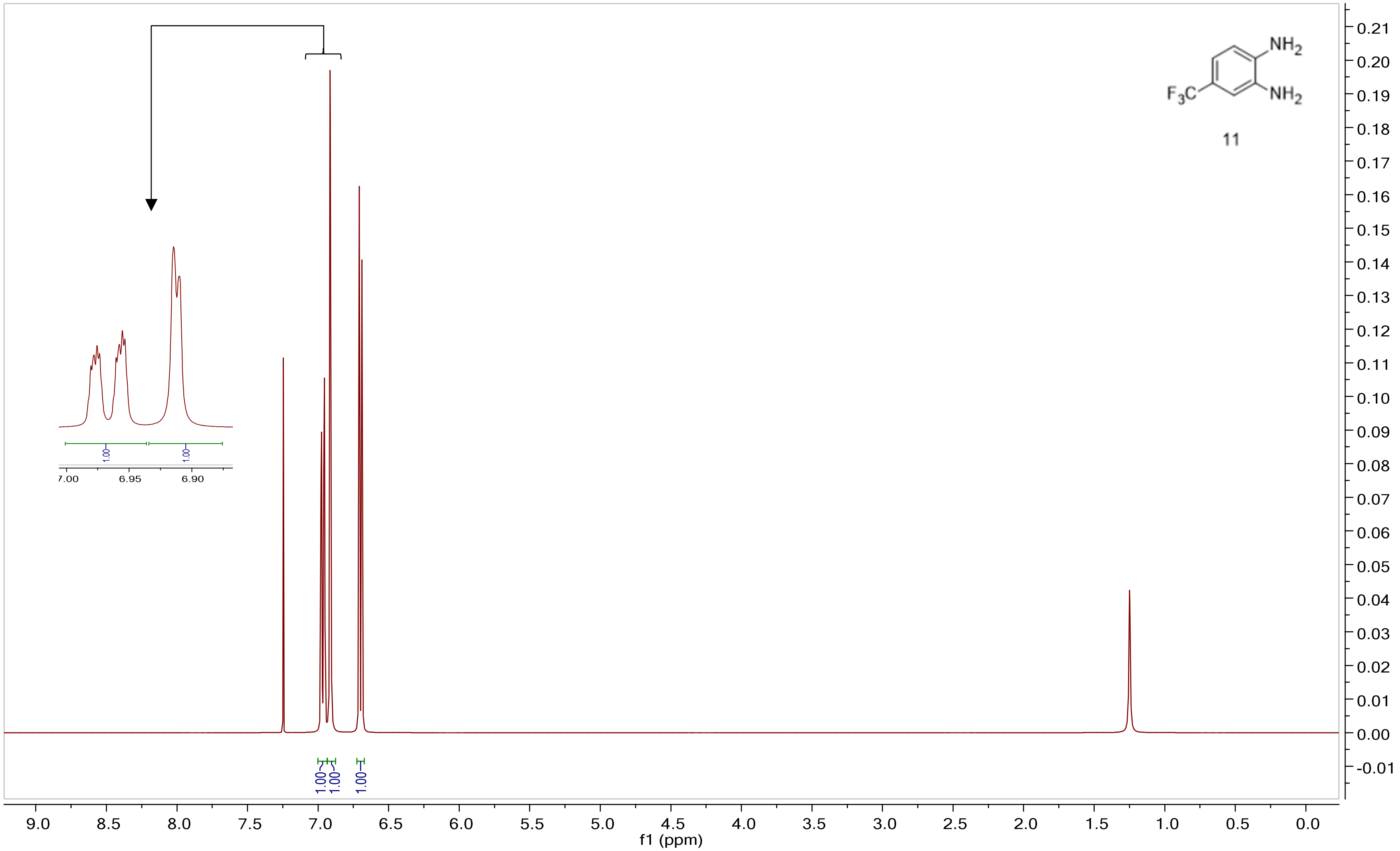

S21. ^{1}H NMR spectrum of analog **11**

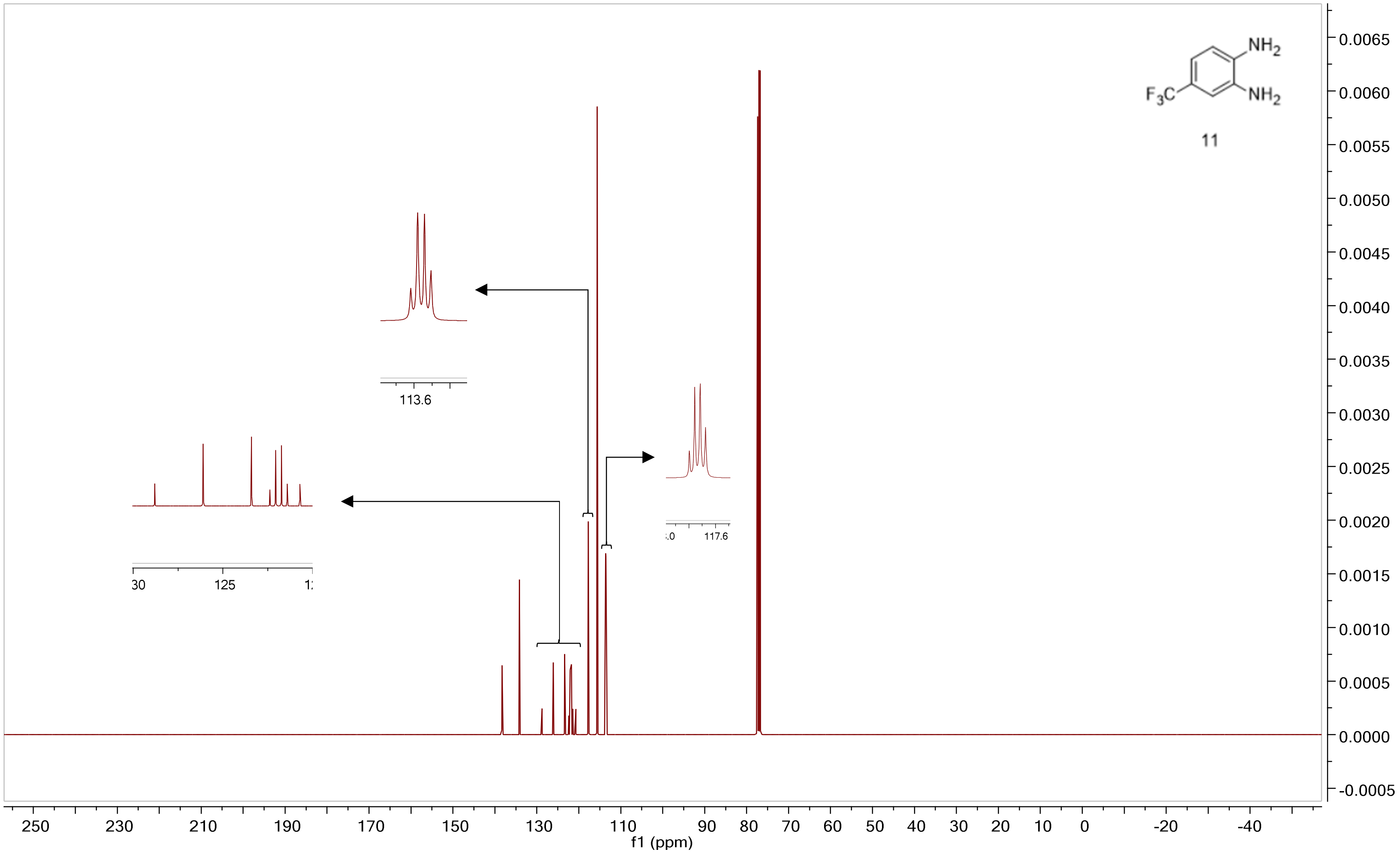

S22. ^{13}C NMR spectrum of analog **11**

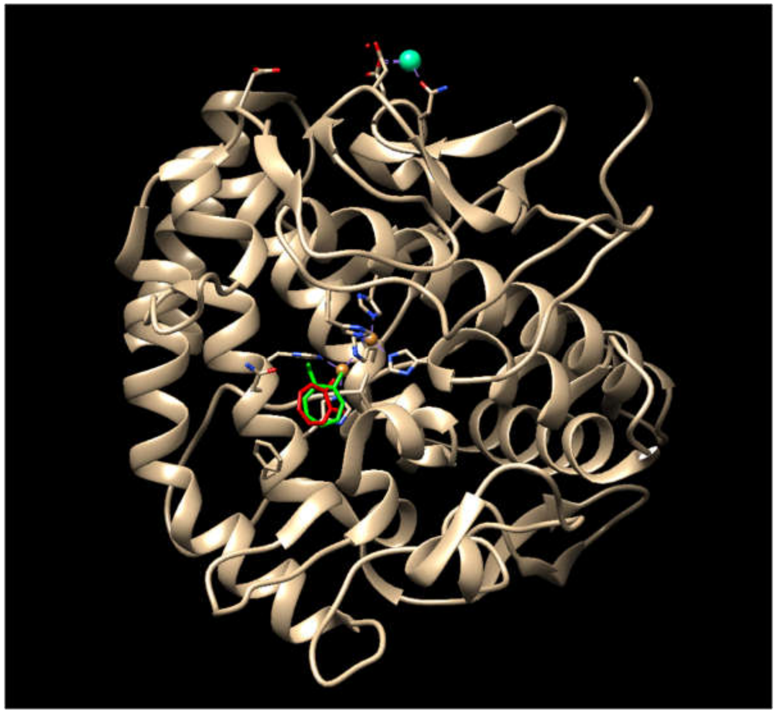

S23. Alignment of the re-docked ligand (green) and co-crystallized ligand (red) with the 2Y9X protein

The validation of the docking procedure was carried out by redocking the co-crystallized tropolone into the tyrosinase active site. In the redocking results, it was found that the re-docked tropolone reproduced the binding pose with a binding affinity of −5.8 kcal/mol. The RMSD of the co-crystallized and experimental poses was analyzed, and the RMSD value was 2.2 Å. These results suggest that the docking simulation could properly accommodate the crystallized ligand.
